# Decision-Making Deficits Associated with Amyloidosis in Lewy Body Disorders

**DOI:** 10.3389/fnhum.2016.00693

**Published:** 2017-01-11

**Authors:** Nicola Spotorno, Corey T. McMillan, David J. Irwin, Robin Clark, Edward B. Lee, John Q. Trojanowski, Daniel Weintraub, Murray Grossman

**Affiliations:** ^1^Penn Frontotemporal Degeneration Center, University of Pennsylvania Perelman School of MedicinePhiladelphia, PA, USA; ^2^Brain Plasticity and Neurodegeneration Group, German Center for Neurodegenerative Diseases (DZNE)Magdeburg, Germany; ^3^Department of Linguistics, University of PennsylvaniaPhiladelphia, PA, USA; ^4^Department of Pathology, Laboratory Medicine and the Center for Neurodegenerative Disease Research, University of Pennsylvania Perelman School of MedicinePhiladelphia, PA, USA; ^5^Department of Psychiatry, University of Pennsylvania Perelman School of MedicinePhiladelphia, PA, USA

**Keywords:** lewy bodies, amyloidosis, decision making, biomarkers, neuroimaging

## Abstract

**Background:** Lewy body disorders (LBD) are clinical syndromes characterized by pathological inclusions containing α-synuclein. Cognitive deficits are common or diagnostic in LBD, and may be associated with the presence of beta-amyloid (Aβ), which is a hallmark histopathologic abnormality characteristic of Alzheimer's disease (AD) that can also co-occur with LBD.

**Objective:** In the present study we evaluated whether social decision-making difficulties in LBD are associated with Aβ burden.

**Methods:** Decision-making abilities were measured with a simple, untimed, behavioral task previously validated in patients with behavioral variant frontotemporal dementia, and performance was related to gray matter atrophy on MRI. Aβ burden was assessed by examination of cerebrospinal fluid (CSF) level of Aβ_1−42_ and by autopsy confirmation in a subgroup of patients.

**Results:** The results revealed that LBD patients with evidence of Aβ have reduced social decision-making abilities compared to patients with no evidence of Aβ. The imaging analysis related greater decision-making difficulty in Aβ-positive patients in respect to Aβ-negative patients to gray matter atrophy in medial orbitofrontal. This region is a critical node of a decision-making network as well as a region previously associated with comorbid α-synuclein and Aβ in LBD.

**Conclusions:** These preliminary findings suggest that cognitive difficulties in LBD extend to include deficits in social decision-making and that this may be related to the presence of Aβ.

## Introduction

Lewy body disorders (LBD) encompass a range of neurodegenerative clinical conditions including Parkinson's disease (PD), Parkinson's disease dementia (PDD) and dementia with Lewy bodies (DLB) that share a common pathological substrate. Specifically, LBD is characterized by pathological inclusions containing the synaptic protein α-synuclein (α-syn) in the cell bodies and processes of surviving neurons (known as Lewy bodies and Lewy neurites, respectively; Goedert et al., 2012; Irwin et al., 2013). Cognitive deficits are common in LBD. Roughly 80% of PD patients eventually evolve over several years to PDD (Emre et al., 2007; Halliday et al., 2008) and, as cognitive deficits are emerging, PD patients can meet criteria for mild cognitive impairment (PD-MCI; Litvan et al., 2012). In other patients, the time between the onset of Parkinsonism and dementia is observed to be within 1 year, or dementia may be present prior to the onset of motor features. These patients are diagnosed with dementia with Lewy bodies (DLB; McKeith et al., 2005; McKeith, 2006; Lippa et al., 2007). Autopsy studies of DLB patients often show that α-syn is not the only histopathologic abnormality that is observed, with several studies also observing co-occurring histopathologic features of Alzheimer's disease (AD). This includes the presence of beta-amyloid (Aβ) plaques and neurofibrillary tangles composed of tau (Kotzbauer et al., 2012). While Aβ is often associated with DLB (Lippa et al., 2007), there is also considerable evidence that Aβ is found in a significant percentage of patients with PDD (Hurtig et al., 2000; Irwin et al., 2013) and recent evidence suggests that over 15% of *de novo* PD patients have evidence of Aβ (McMillan and Wolk, 2016).

The range of cognitive deficits observed in LBD includes deficits in visuoconstructional, episodic memory, and language domains. Executive deficits are arguably the most common cognitive impairment observed in the LBD spectrum (Levin et al., 1991; Rosenthal et al., 2010; Dirnberger and Jahanshahi, 2013). This is closely aligned with difficulties in decision-making and social functioning (Bodden et al., 2010; Djamshidian et al., 2014), and these deficits can have a profound impact on patients' daily lives (Lo et al., 2009; Rosenthal et al., 2010).

With the emergence of potential disease-modifying treatments, there is considerable interest in defining more specifically the neurobiologic basis for cognitive difficulties, and developing inexpensive, non-invasive screening tools that can both help improve pathology-associated diagnosis and serve as a validated, repeatable endpoint in an intervention study. One potential strategy would involve associating specific cognitive difficulties with a particular histopathologic abnormality. For instance, elevated amyloid retention on PET imaging appears to be associated with cognitive deficits in patients with DLB (Gomperts et al., 2012), PD-MCI (Petrou et al., 2012) and also in non-demented PD patients (Gomperts et al., 2013). However, some studies (Jokinen et al., 2010) found no association between amyloid PET uptake and cognitive functioning in LBD. There is a close relationship between CSF and PET imaging measures of amyloid (Landau et al., 2013; Palmqvist et al., 2015), and a related strategy demonstrates an association between cognitive difficulty and the cerebrospinal fluid (CSF) level of Aβ_1−42_ (Alves et al., 2010; McMillan and Wolk, 2016). In particular CSF Aβ_1−42_ in early PD has been associated with increased frontal lobe dysfunctions including executive impairments relative to individuals with early PD and no evidence of Aβ pathology (McMillan and Wolk, 2016). Given the inconsistent results in these approaches, it would be valuable to have independent validation of the association between cognitive difficulty and amyloid in LBD that might come in part from relating amyloid to a specific anatomic locus of a cognitive deficit.

In the present study, we sought to provide preliminary evidence of the impact of amyloid pathology on a measure of social decision-making, which involves prefrontal networks, in patients with LBD.

The task we employed was developed to investigate decision-making during social coordination. Social decisions often require two individuals to converge on the same thought despite the absence of explicit information. In game-theoretic terms, this ability of getting on the same page' without a direct exchange of information is called a “coordination game.” In certain coordination games, players can only reach the solution by inferring implicit mutual knowledge between the participants. In other words, players must use a “focal point,” defined as a salient source of information known to both players, which transcends the mathematical structure of the game (Schelling, 1969). In a seminal study, Mehta et al. (1994) asked participants to select a boy's name under two conditions: “picking” (pick any name) and “coordination” (pick the same name as a random partner). The name “John” was only given in 9% of “picking” responses but was given in 50% of “coordination” responses. Thus, due to the belief that a random partner would have in mind a name that many believe is common, participants in this study established the common name “John” as a focal point. Notably, this paradigm is simple, untimed, requires a minimal response of one word, yet involves an essential decision-making process that we use all the time.

Previous work from our group has employed a modified version of Mehta et al. paradigm to investigate decision-making abilities during social coordination in patients affected by behavioral variant frontotemporal degeneration (bvFTD) (McMillan et al., 2012). This is a neurodegenerative condition that compromises prefrontal functioning with minimal impact on language (Rascovsky et al., 2011). This work showed that bvFTD patients are impaired in establishing a focal point, and associated these difficulties with cortical thinning in a network of prefrontal regions including ventromedial, ventrolateral, and dorsolateral prefrontal cortex as well as medial orbitofrontal cortex. These prefrontal areas were also previously associated with Aβ in LBD patients (McMillan and Wolk, 2016).

Patients were divided into Aβ-positive and Aβ-negative groups on the basis of a validated CSF Aβ_1−42_ level (Shaw et al., 2009) or autopsy confirmed amyloidosis (McKeith, 2006). A subgroup of participants also had high-resolution MRI scans to correlate behavioral performance with regional decrease in gray matter (GM) density. We hypothesized that decision-making difficulties in LBD patients would be more pronounced in individuals with a significant Aβ burden, and that this would be associated with a reduction in GM density in prefrontal regions contributing to decision-making.

## Materials and methods

### Participants

From a cohort of 77 patients with a LBD disorder evaluated with this decision-making paradigm, we selected 37 patients with a clinical diagnosis of a LBD disorder who also underwent the CSF protocol (*N* = 32) or for which autopsy examination was available (*N* = 5). This group included 8 patients with PD, 15 patients with PD-MCI, 6 patients with PDD, and 8 patients with DLB. Patients were diagnosed by neurologists using published consensus criteria (Hughes et al., 1992; McKeith, 2006; Emre et al., 2007; Litvan et al., 2012). Patients with other neurodegenerative diseases were excluded. Patients with a neurologic condition such as stroke or hydrocephalus, a primary psychiatric disorder, or a medical condition causing cognitive difficulty were also excluded. We also assessed a group of 30 community-dwelling healthy seniors (see Table 1 for participants' demographic information and statistics). All subjects participated in an informed consent procedure approved by the University of Pennsylvania Institutional Review Board.

**Table 1:** **Mean (±SEM) demographic data for the patients and the healthy seniors groups (A), Mean (±SEM) demographic data for ***amyloid-negative*** and ***amyloid-positive*** groups (B)**.

**Demographic/clinical measure**	**Patients (*N* = 37)**	**Healthy seniors (*N* = 30)**
**(A)**		
Age (years)^*^	70 (1)	65 (1)
Education (years)	15.8 (0.4)	14.9 (0.4)
Gender (female/male)	12/25	9/21
Ethnicity (Cw/BA/M)^*^^1^	36/1/0	22/7/1
MMSE score (max = 30)^*^	26.5 (0.7)	29.1 (0.1)
Disease duration (years)	10 (1)	–
Test–CSF collection interval (years)^2^	2.1 (0.3)	–
Test–death interval (years)^3^	2.6 (0.2)	–
***Demographic/clinical measure***	***Amyloid-negative (N*** **= 25*)***	***Amyloid-positive (N*** **= 12*)***
**(B)**		
Age (years)	69 (1)	70 (3)
Education (years)^*^	16.8 (0.5)	14.1 (0.7)
Gender (female/male)	8/17	4/8
MMSE score (max = 30)^*^	27.4 (0.6)	24.6 (1.6)
Disease duration (years)	11 (1)	7 (1)
Number PD cases	7	1
Number PD-MCI cases	11	4
Number PDD cases	4	2
Number DLB	3	5
CSF-date to behavioral test (months)	27 (4)	20 (5)

* Significant difference between two groups.

1 *Cw, Caucasian-white; BA, Black-American; M, mixed heritage*.

2 *The data refers to the group of 32 patients for which a CSF sample was available*.

3 *The data refers to the group of 5 patients with neuropathological examination*.

***(A)** Patients and healthy seniors differed in age (Wilcoxon W = 359; p < 0.05), MMSE score (Wilcoxon W = 809; p < 0.01) and ethnicity (comparison between Caucasian-White American and Black-Americans: X^2^ = 5.1447; p < 0.05) while the two groups did not differ in genders distribution (X^2^ < 0.0001; p > 0.9), and level of education (Wilcoxon W = 440.5; p > 0.1). **(B)** Amyloid-negative and amyloid-positive patients did not differed in age (Wilcoxon W = 136.5 p > 0.6), in gender distribution (X^2^ < 0.0001; p > 0.9) and in the disease duration (Wilcoxon W = 183; p > 0.1) while the two groups differed in the level of education (Wilcoxon W = 234; p < 0.01) and in their MMSE score (Wilcoxon W = 788; p < 0.05)*.

### Behavioral procedure and analysis

Participants were presented with 10 questions (e.g., “Tell me the name of any _____”) probing a familiar semantic category (e.g., “fabric,” “boy's name,” “supermarket item”), as described previously (McMillan et al., 2012). In the *Survey* condition, participants were told that we were conducting a survey and that they could answer each question however they wished, allowing us to obtain baseline performance. They were also informed that the survey would not be followed by any memory test. Following a 25-min delay during which other cognitive measures were administered, each question was repeated in the *Coordination* condition. Here, participants were instructed to provide the answer that they thought another participant in the same survey would have provided. Participants were always presented the *Survey* condition first and then the *Coordination* condition, in an effort to obscure the decision-making nature of the task.

Responses were quantified in both the *Survey* and *Coordination* condition using a “Coordination Index” based on the frequency of occurrence of each response provided during the *Coordination* condition in the pool of healthy seniors. To every response that was not provided by the group of healthy seniors in the *Coordination condition* was given a frequency of zero. For example, the pair of answers (*survey* and *coordination* condition) “horse—chicken” in the category *animals* obtained the scores: 0–1, while the pair “horse—cat” obtained the score: 0–8 (see McMillan et al., 2012 for further details). The scores of the single categories were averaged in order to obtain two global scores for each participant: one for the *Survey condition* and one for the *Coordination condition*.

### CSF analysis

CSF samples were obtained by lumbar puncture using a 22-gauge spinal needle as described in the Alzheimer's Disease Neuroimaging Initiative (ADNI) procedures manual (http://www.adni-info.org/). CSF was divided into aliquots (0.25 mL) and stored in bar code–labeled polypropylene vials at -80°C. Aβ_1−42_ was measured using the multiplex xMAP Luminex platform (Luminex Corp, Austin, TX) with Innogenetics (INNO-BIA AlzBio3; Ghent, Belgium; for research use only reagents) immunoassay kit–based reagents (Shaw et al., 2009). Full details for the combination of immunoassay reagents and analytical platform employed in the present study are explained elsewhere (Irwin et al., 2012). Reliability studies (http://www.adni-info.org) show that the reproducibility for this biomarker varies by < 10%. In line with previous studies (Shaw et al., 2009), CSF Aβ_1−42_ was analyzed as a binary measure employing a level of ≤ 192 pg/mL as a cutoff (hereafter, *amyloid-positive* ≤ 192 pg/mL; *amyloid-negative* ≥192 pg/mL). This criterion allowed us to identify two subgroups of patients: *amyloid-positive* patients (≤ 192 pg/mL, *n* = 12) and *amyloid-negative* patients (≥192 pg/mL, *n* = 25).

### Autopsy procedure

A subset of PD spectrum patients had ante mortem MRI and cognitive evaluations with autopsy confirmation of amyloidosis (*n* = 5), and thus these patients were included for study. Briefly, fresh tissue was dissected at autopsy, fixed, processed, and stained according to standard procedures described elsewhere (Toledo et al., 2011). A neuropathological diagnosis was performed by an expert neuropathologist (EBL, JQT) using standard criteria (McKeith et al., 2005; Montine et al., 2012). Cases were classified into *amyloid-positive* and *amyloid-negative* groups based evidences of comorbid Alzheimer's disease: none or low probability (*amyloid-negative*), intermediate or high probability (*amyloid-positive*). Table 2 shows neuropathological group data.

**Table 2:** **Neuropathological information and Alzheimer's disease neuropathological diagnosis criteria on 5 patients for whom autopsy examination was available**.

**Patient**	**PMI**	**Brian volume**	**Clinical phenotype**	**Probability-AD-pathology**	**Study-specific group**
N1	7	1079	DLB	Low	*Amyloid-negative*
N2	23.5	1220	PDD	Low	*Amyloid-negative*
N3	10	1390	PDD	None	*Amyloid-negative*
N4	14	1322	PDD	Intermediate	*Amyloid-positive*
N5	9.5	1258	PDD	High	*Amyloid-positive*

### Imaging procedure and analysis

High-resolution volumetric T1-weighted MRI was obtained within 9 months of behavioral testing (mean ± SEM = 3.5 ± 0.6) for 26 patients (17 *amyloid-negative* and 9 *amyloid-positive*). These patients matched the overall group of patients in age, education, MMSE and gender. A Shapiro test revealed that the values for age, education and MMSE were not normally distributed, thus nonparametric tests were employed (age: Wilcoxon *W* = 510, *p* > 0.6; education: Wilcoxon *W* = 473.5, *p* > 0.9; MMSE: Wilcoxon *W* = 486.5, *p* > 0.9; gender: *X*^2^ < 0.001, *p* > 0.9; disease duration: Wilcoxon *W* = 507.5, *p* > 0.2). Reasons for exclusion from the MRI study included issues related to health and safety (e.g., metallic implants, shrapnel, claustrophobia), intercurrent medical illness, and lack of interest in participating in an imaging study. MRI volumes were acquired using an MPRAGE sequence from a SIEMENS 3.0T Trio scanner with an 8-channel head coil and the following acquisition parameters: repetition time = 1620 msec; echo time = 3.87 msec; slice thickness = 1.0 mm; flip angle = 15°; matrix = 192 × 256, and in-plane resolution = 1.0 × 1.0 mm. Whole-brain MRI volumes were preprocessed using PipeDream (https://sourceforge.net/projects/neuropipedream/) and Advanced Normalization Tools (http://www.picsl.upenn.edu/ANTS/) using a procedure described previously (Avants et al., 2008; Klein et al., 2010). Resulting images were warped into MNI space, smoothed using a 4 mm full-width half-maximum Gaussian kernel, and downsampled to 2 mm resolution.

Permutation-based imaging analyses were performed with threshold-free cluster enhancement (TFCE; Smith and Nichols, 2009) using the randomize tool in FSL (http://fsl.fmrib.ox.ac.uk/fsl/fslwiki). GM density was compared in patients relative to healthy seniors who were part of an independent group of 32 healthy seniors with imaging who matched the patient group for education (Wilcoxon *W* = 374, *p* > 0.1), age (Wilcoxon *W* = 330.5, *p* > 0.1) and gender (*X*^2^ = 0.19, *p* > 0.7). Considering that the main purpose of the imaging analysis is to regress the behavioral performance against GM density in the patients group and that the scans of healthy seniors have been employed only to determine the atrophy mask, the use of an independent group of healthy seniors does not compromise the quality of the analysis (for a similar strategy see e.g., Grossman et al., 2004; Henry et al., 2008; Healey et al., 2015; Ash et al., 2016). A *t*-test analysis was run with 10,000 permutations and restricted to voxels containing GM using an explicit mask generated from the average GM density map of all participants. We report clusters that survived a threshold of *p* < 0.05 with family-wise error (FWE) correction for multiple comparisons and contained a minimum of 100 adjacent voxels.

To relate behavioral performance to regions of significant GM disease, we used the randomize tool of FSL with TFCE technique as described above. We run a regression analysis between patients' performance in the task and the GM density in regions of the brain showing decreasing GM density with respect to the pool of healthy seniors in order to focus our analysis to regions probably affected by the disease state. The regression model included the Aβ status (positive or negative) as a variable of interest. Permutations were run exhaustively up to a maximum of 10,000. We report clusters surviving a height threshold of *p* < 0.005 (uncorrected) and a minimum of 20 adjacent voxels.

## Results

### Behavioral results

**Figure 1:**
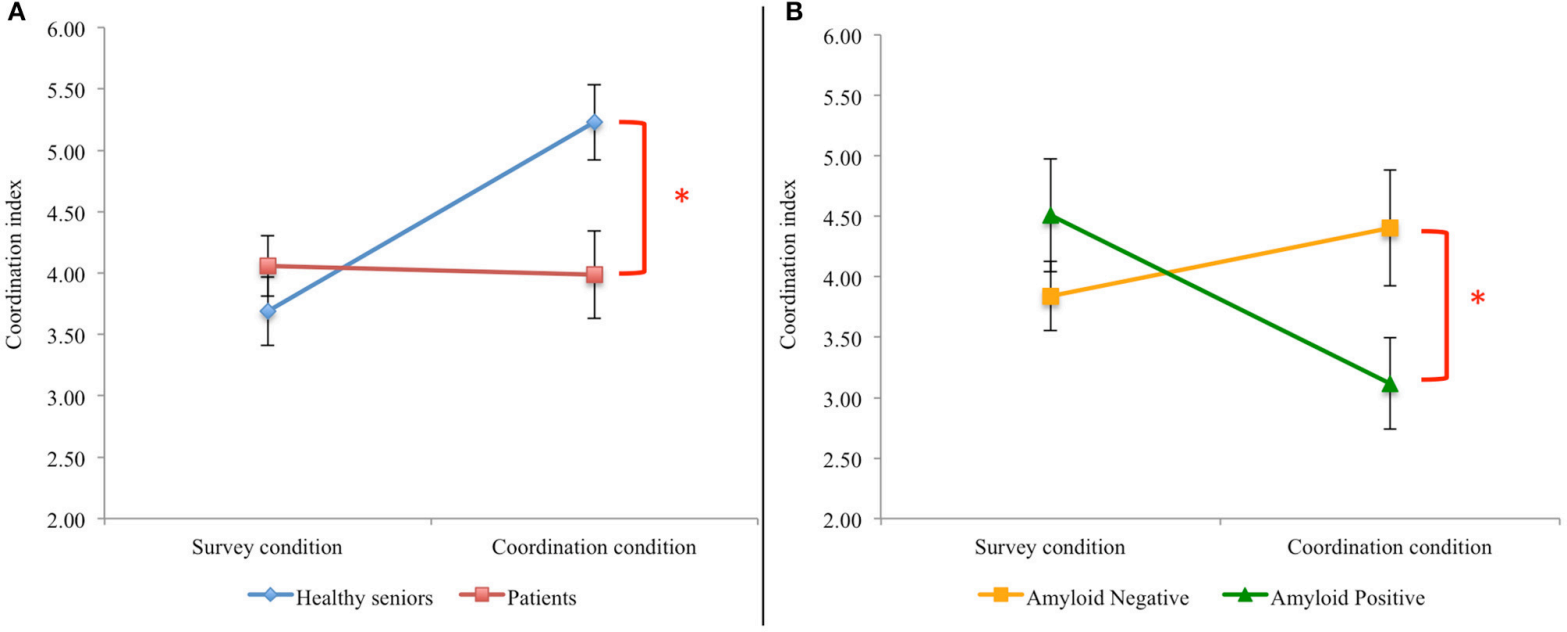
**Behavioral results**. The coordination index represent the frequency with which a specific answer has been provided by the pool of healthy seniors. The error bars represent the standard deviation of the mean while the square brackets and the stars display the significant effect of interaction. **(A)** Comparison between the performance of healthy seniors and the performance of the global group of patients. **(B)** Comparison between the performance of *amyloid-positive* patients and the performance of *amyloid-negative* patients.

A regression model was built to test if the performance of the group of LBD patients (*N* = 37) differed from the performance of the healthy seniors (*N* = 30). The model also included age as covariate. The results showed a significant main effect of the Experimental condition (*Survey* or *Coordination: t* = −3.57, *p* < 0.001, Cohen's *d* = −0.6), a main effect of Group (healthy seniors or patients: *t* = −2.12, *p* < 0.05, Cohen's *d* = −0.4), and an interaction between the two variables (Experimental condition^*^Group: *t* = 2.77, *p* < 0.01, Cohen's *d* = 0.5). Age also reached a significant threshold (*t* = −3.83, *p* < 0.001, Cohen's *d* = −0.7). However, a follow up analysis that included Experimental condition^*^Age in the model revealed no significant effect of the interaction between the two variables (*t* = 0.548; *p* > 0.5, Cohen's *d* = 0.1). These findings, summarized in Figure 1A, showed that LBD patients are significantly impaired in their social decision-making ability compared with healthy seniors.

We have also investigated the influence of being part of different ethnical groups on the behavioral performance in our task. The majority of our group of participants was composed by Caucasian-white American people (58 participants out of 67). In the group of healthy seniors 7 participants were Black-Americans and 1 had mixed heritage. In the group of patients only 1 participant was Black-American. An exploratory regression model in which we added *ethnicity* as a regressor, but excluded the single participant with mixed heritage, showed that this variable did not explain a significant amount of variance (*t* = 1.282; *p* > 0.2, Cohen's *d* = 0.2). A follow up analysis restricted to healthy seniors, among whom there was the largest subgroup of Black-Americans, revealed no effect of *ethnicity* on the performance in the task [*ethnicity*: *t* = −0.888; *p* > 3, Cohen's *d* = −0.2; ethnicity^*^experimental condition (*survey*—*coordination*): *t* = 1.620; *p* > 0.1, Cohen's *d* = 0.4].

CSF analysis and autopsy exam identified that 32% of LBD patients had evidence of Aβ, consistent with prior reports (Hurtig et al., 2000; Irwin et al., 2013; McMillan and Wolk, 2016; see Table 1 for further details). Another regression model was built to test the differences between the *amyloid-positive* (*N* = 12) and *amyloid-negative* (*N* = 25) groups of patients. The two groups of patients differ in their average level of education and MMSE score, thus both scores were included as covariates in the model as well as disease duration. The results revealed no main effect of the experimental condition (*t* = −0.80, *p* > 0.4, Cohen's *d* = −0.2) or patient group (*t* = −1.54, *p* > 0.1, Cohen's *d* = −0.4), but a significant interaction effect between the experimental condition and the patient group was found (*t* = 2.01, *p* < 0.05, Cohen's *d* = 0.5). None of the covariates explained a significant amount of variance (MMSE: *t* = 1.44, *p* > 0.1, Cohen's *d* = 0.4; disease duration *t* = −0.74, *p* > 0.4, Cohen's *d* = −0.2; level of education: *t* = −0.61, *p* > 0.5, Cohen's *d* = −0.1). As shown in Figure 1B, *amyloid-negative* patients tended to choose uncommon referents in the *survey condition* but then shifted to more common referents in the *coordination condition*. By comparison, *amyloid-positive* patients tended to choose more common referents in the *survey condition* but did not coordinate in the *coordination condition* and tended to produce less common referents.

To test whether the presence of dementia could explain coordination difficulty, we also examined performance in patients diagnosed clinically with dementia (PDD or DLB, *N* = 14) compared with patients without dementia (PD or PD-MCI patients; *N* = 23). Although the sample is small, a regression analysis showed no significant main effects or interaction effect [phenotype (*Clinically demented patients* or *non-demented patients*): *t* = 0.33, *p* > 0.7, Cohen's *d* = 0.1; Experimental variable (*Survey* or *Coordination*): *t* = 0.93, *p* > 0.3, Cohen's *d* = 0.2; interaction: *t* = −1.51, *p* > 0.2, Cohen's *d* = −0.4].

### Imaging results

**Figure 2:**
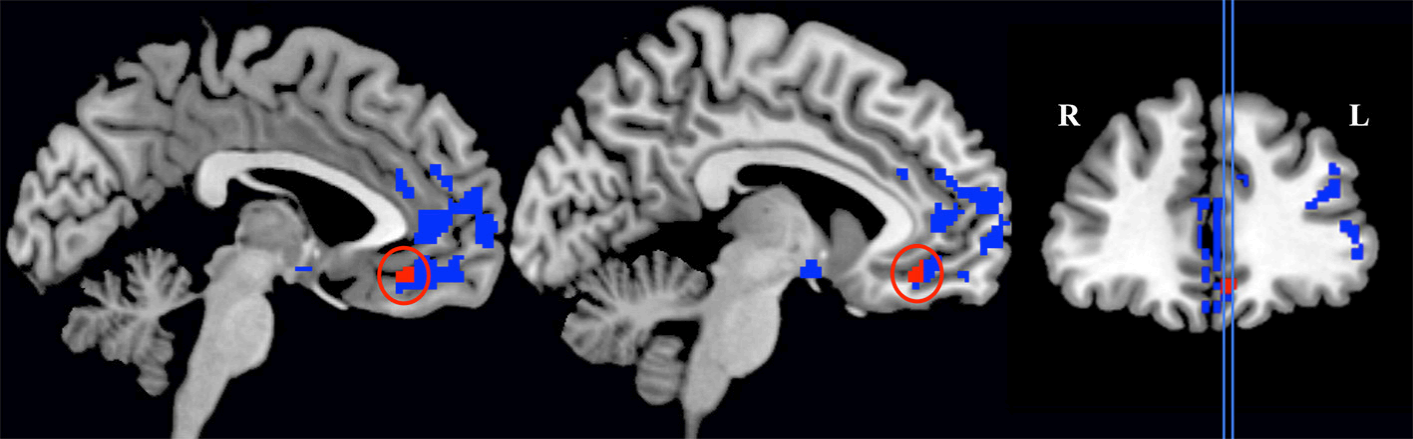
**Significant gray matter atrophy in the global group of patients (this includes areas colored both in blue and in red) and regions (in red only) that showed a significant interaction between Experimental conditions (***Survey*** and ***Coordination*** conditions) and the patients' amyloid status (***amyloid-positive*** and ***amyloid-negative*** patients)**.

We first compared GM density in patients versus the group of healthy seniors. The results revealed significant atrophy in patients bilaterally in lateral prefrontal cortex, medial orbitofrontal cortex, middle temporal regions, and insular cortex, as well as significant atrophy in the right fusiform gyrus. Atrophy coordinates are summarized in Table 3. A regression analysis with Aβ status (positive or negative) and behavioral performance (*Survey* or *Coordination* condition) as regressors of interest revealed a significant interaction between the two regressors in the medial orbitofrontal cortex (mOFC; BA 11). In other words (see Figure 2), difficulty with social decision-making in the *amyloid-positive* group was associated with gray matter atrophy in mOFC. This region as been previously linked to social decision-making and, in particular to the neural underpinnings of task employed we employed here (Murray et al., 2007; Wallis, 2007; McMillan et al., 2012). This region also shows increased burden of Aβ in autopsy studies of LBD (McMillan and Wolk, 2016).

**Table 3:** **Regions with reduced gray matter density in the patients' cohort respect to the group of healthy seniors (A), Results of the regression analysis showing a significant interaction between Aβ status (positive or negative) and behavioral performance (***Survey*** or ***Coordination*** condition) (B)**.

**Patients < healthy seniors**	**L/R**	**N^o^ voxels**	***t-*****value (max)**	***p*****-value**	**MNI Coordinates**		
					**x**	**y**	**z**
**(A) REDUCED GM (BA)**							
Middle tempral gyrus, superior temporal gyrus (21–22) and insular cortex	L	6097	5.21	<0.01	−64	−30	2
Anterior cingulate cortex and medial prefrontal cortex (10–11)	L/R	1326	5.03	<0.05	10	48	6
Fusiform gyrus	R	425	4.03	<0.05	30	−8	−38
Middle temporal gyrus (21)	R	245	4.14	<0.05	68	−32	−12
Superior temporal gyrus (22)	R	175	4.01	<0.05	68	−32	18
Insular cortex	L	149	4.83	<0.05	44	2	−2
**(B) RESULTS OF THE REGRESSION ANALYSIS IN THE LBD COHORT (BA)**							
Medial orbitofrontal cortex (11)	L/R	21	3.73	<0.005	−6	36	−12

*BA, Brodmann area; MNI, Montreal Neurological Institute*.

## Discussion

In the present study we examined patients with a LBD to investigate the extent to which concomitant amyloid burden worsens the decision-making profile of these patients. We employed a task previously studied in neurodegenerative disease that depends on a decision-making network in prefrontal cortex. The results revealed that *amyloid-positive* patients have reduced decision-making abilities relative to *amyloid-negative* patients. The imaging analysis related significant decision-making difficulty in *amyloid-positive* patients to atrophy in medial orbitofrontal cortex, a region that has been previously implicated in decision-making. In particular, previous work has implicated the same region in perspective-taking during social interactions (Mehta et al., 1994; McMillan et al., 2012; Healey et al., 2015). McMillan et al. (2012), for example, showed that poor performance in the same social-coordination task that was employed here, related with cortical thinning in the mOFC in bvFTD patients. In mOFC has been implicated as well in processes like encoding stimulus-outcome contingencies, assessing potential risk and reward, and performing tasks involving reversal learning or response inhibition (Murray et al., 2007; Viskontas et al., 2007; Wallis, 2007).

Many studies have documented the accumulation of amyloid pathology in a large percentage of patients with LBD (Hurtig et al., 2000; Siderowf et al., 2010; Irwin et al., 2012; Kotzbauer et al., 2012). This includes patients with the clinical diagnosis of DLB and PDD (Irwin et al., 2012; Kotzbauer et al., 2012), as well as patients with PD-MCI and even in patients with early, untreated PD who have no apparent cognitive impairment (Alves et al., 2010). The consequences of accumulating amyloid for cognitive impairment in LBD have been unclear. While some studies have reported an association between cognitive impairment and amyloid accumulation in LBD (Alves et al., 2010; Siderowf et al., 2010; Petrou et al., 2012), there are several potential sources of confound. On the one hand, some of the controversy surrounding the extent of cognitive impairment in LBD may be due in part to due to the neuropsychological tests that have been used as markers of cognitive impairment, since many studies have focused on non-specific cognitive measures such as MoCA, MMSE, or the Mattis Dementia Rating Scale. For example, these inconsistently emphasize a variety of cognitive skills that may or may not be sensitive to the cognitive deficits in LBD. It is for this reason that we decided to examine a specific measure of decision-making where we could clearly determine whether a patient has a deficit. This measure has minimal resource demands since it requires an untimed, single word response to a simple question.

Another potential confound has to do with the manner in which amyloid accumulation is ascertained. Previous studies have used either amyloid PET or CSF Aβ_1−42_ as a proxy for brain amyloid. While each appears to be a valid measure of amyloid, there has been some concern about validating the relationship between amyloid and cognitive dysfunction, particularly given the inconsistent presence of amyloid at autopsy in LBD. In this study, amyloid status was assessed in each individual. We used CSF level of amyloid as a surrogate reflecting amyloid burden in the brain, in addition to direct post-mortem evaluation in a subset of cases. We found that CNS amyloidosis is present in 32% of patients participating in this study, and CSF amyloid levels were reduced in all clinical subgroups of LBD patients. The results of the present study cannot be easily attributed to a general decrement in cognitive performance in the *amyloid-positive* group because *amyloid-positive* patients showed worse performance than *amyloid-negative* patients even after including MMSE, level of education, and disease duration in the regression model.

Several shortcomings should be taken into consideration in evaluating the results of the present work. Although we used patients from a deeply endophenotyped dataset that requires critical collection of multiple forms of data, the number of patients is relatively small. Thus, the inferences we derive from the present analysis must be verified in future studies employing a larger cohort. Additional studies with different behavioral protocols are needed to evaluate the extent to which amyloid burden contributes to decision-making as well as to other cognitive domains that depend on other neural networks. While amyloid burden is widely distributed throughout the gray matter in these patients, recent work has associated amyloid burden in specific brain regions with worse cognitive functioning that depends in part on the target regions. For example, early evidence of amyloid in *de novo* PD has been associated with impaired verbal fluency and disease in regions similar to those observed in the current study (e.g., mOFC) (McMillan and Wolk, 2016). We cannot entirely rule out that possible executive limitations, such as the ones tested by a verbal fluency test, had an impact on the performance in our task. However, it can be argued that decision-making processes are partially rooted in executive resources and than the two cannot be entirely disentangled. More precisely McMillan et al. (2012) have found that, in a cohort of bvFTD patients, performance in the very same task employed here correlated with the scores in the Visual-Verbal test (Feldman and Drawgow, 1960), which test mental flexibility. In addition to that, it is possible that social coordination is partially based on probability-estimation, which is also heavily dependent upon executive resources such as mental flexibility and working memory (see e.g., Brand et al., 2005, 2007; Del Missier et al., 2010). However, future studies must address questions about the specificity of our task and his redundancy in respect with other exiting tasks like verbal fluency or MMSE as well as the relevance of specific facets of the executive functions for the execution of the social-coordination task. At the moment we would just like to point out that a task that test a multidimensional process such as social-coordination can still be of clinical interest because it might provide a more “realistic” testing ground in respect to standard neuropsychological tests.

Another possible shortcoming of the present work is the heterogeneity of the clinical cohort. Future works should investigate the impact of the different clinical phenotypes within the LBD spectrum to the interaction between amyloid burden and decision-making abilities. Cognitive deficits can vary drastically among phenotypes but our claims are restricted to a single decision-making test that has been administered to all the participants and a regression analysis showed no significant interaction between clinical phenotype and the performance in the task. In addition to that, significant amyloid burden can be found in LBD patients across the boarders of clinical phenotypes (see e.g., Irwin et al., 2013; McMillan and Wolk, 2016).

We acknowledge also that there was a great variability in the interval between behavioral testing and the collection of CSF as well as in the interval between behavioral testing and death in our sample (see Table 1). Although we cannot completely rule out that the delay between behavioral testing and both CSF and neuropathological examination could be a confounding factor, previous studies have shown that, at least in Alzheimer's disease, the level of beta-amyloid in the brain reaches a plato early on and stays almost stable over the course of disease progression (see e.g., Engler et al., 2006; Sperling et al., 2011). Therefore, the estimation of beta-amyloid level via CSF or neuropatholagical examination can be considered as a stable value.

It must be also noticed that the age of the participants explains a significant amount of variance in our first regression model. However, a follow up analysis that included the interaction between the experimental condition and age in the model revealed that this term did not explain a significant amount of variance. Moreover, the main focus of our study was the comparison within the patients group and in this scenario the age of the *amyloid-positive* and the age of the *amyloid-negative* patients were tightly matched.

Another possible caveat is that amyloid co-occurs with tau in the form of neurofibrillary tangles in these patients, and we cannot rule out the possibility that tau burden also contributes to decision-making difficulty in these patients. With these caveats in mind, we conclude that the present work provides preliminary evidence that links impaired decision-making to Aβ burden that reflects the accumulation of amyloid in the prefrontal cortex of patients with a LBD.

## Author contributions

NS, CM, and MG were responsible for the conceptualization of the study, analysis and interpretation of the data, and drafting or revising the manuscript. RC was responsible for the conceptualization of the study and for revising the manuscript. DI, EL, and JT were responsible for autopsy examination and revising the manuscript. DW was responsible for conceptualization and revising the manuscript. All the authors have approved the final version of the manuscript.

## Funding

Morris K. Udall Research Center of Excellence NS053488, AG043503, AG10124, AG17586, AG038490, joint support from Alzheimer's Association and Michael J Fox Foundation (BAND-14-338181), the Dana Foundation, and the Wyncote Foundation.

### Conflict of interest statement

The authors declare that the research was conducted in the absence of any commercial or financial relationships that could be construed as a potential conflict of interest.
